# Exciton–exciton annihilation and biexciton stimulated emission in graphene nanoribbons

**DOI:** 10.1038/ncomms11010

**Published:** 2016-03-17

**Authors:** Giancarlo Soavi, Stefano Dal Conte, Cristian Manzoni, Daniele Viola, Akimitsu Narita, Yunbin Hu, Xinliang Feng, Ulrich Hohenester, Elisa Molinari, Deborah Prezzi, Klaus Müllen, Giulio Cerullo

**Affiliations:** 1 Dipartimento di Fisica, Politecnico di Milano, Piazza Leonardo Da Vinci 32, Milano 20133, Italy; 2 Istituto di Fotonica e Nanotecnologie, CNR, Piazza Leonardo Da Vinci 32, Milano 20133, Italy; 3 Max Planck Institute for Polymer Research, Ackermannweg 10, Mainz 55128, Germany; 4 Institute of Physics, University of Graz, Universitätsplatz 5, Graz 8010, Austria; 5 Dipartimento di Scienze Fisiche, Informatiche e Matematiche, Università di Modena e Reggio Emilia, Modena 41125, Italy; 6 Istituto Nanoscienze, CNR, via G. Campi 213/a, Modena 41125, Italy; 7 Present address: Cambridge Graphene Centre, University of Cambridge, Cambridge CB3 0FA, UK

## Abstract

Graphene nanoribbons display extraordinary optical properties due to one-dimensional quantum-confinement, such as width-dependent bandgap and strong electron–hole interactions, responsible for the formation of excitons with extremely high binding energies. Here we use femtosecond transient absorption spectroscopy to explore the ultrafast optical properties of ultranarrow, structurally well-defined graphene nanoribbons as a function of the excitation fluence, and the impact of enhanced Coulomb interaction on their excited states dynamics. We show that in the high-excitation regime biexcitons are formed by nonlinear exciton–exciton annihilation, and that they radiatively recombine via stimulated emission. We obtain a biexciton binding energy of ≈250 meV, in very good agreement with theoretical results from quantum Monte Carlo simulations. These observations pave the way for the application of graphene nanoribbons in photonics and optoelectronics.

## Abstract (web summary)

Graphene nanoribbons confine electrons to just one dimension and this gives rise to strong electron–hole interactions. Here, the authors investigate the creation and recombination of biexcitons in these structures by ultrafast optical pulses using femtosecond transient absorption spectroscopy.

## Graphical abstract

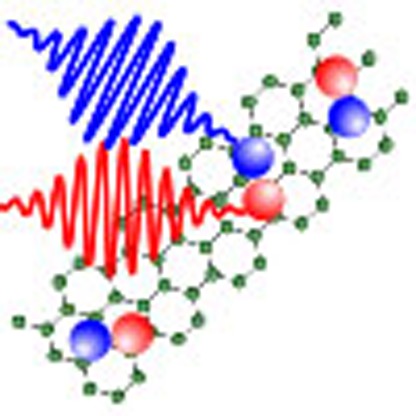

Slicing graphene into nanoribbons (GNRs) allows to open a bandgap in the graphene electronic structure, owing to the quasi-one-dimensional confinement^1^. This has important implications for electronic devices, such as GNR transistors^2^, and forms the basis of the emerging field of graphene nanoplasmonics^3^,^4^. Especially appealing is also the possibility to additionally tailor specific properties through edge-structure engineering, such as magnetic ordering in zigzag terminated GNRs^5^,^6^. Further improvements along these lines are envisaged on the basis of recent advances in fabrication^7^,^8^,^9^,^10^ and processing routes^11^,^12^. In particular, the bottom-up synthesis^7^,^8^ of GNRs based on molecular precursors designed on purpose has proven capable of reaching nanometric widths with atomically precise edges, a regime where the GNR properties are widely tunable^1^. While single-walled carbon nanotubes (SWNTs) cannot be prepared with single chirality and require further processing with surfactants for the sorting^13^,^14^, the bottom-up synthesis directly affords GNRs with a uniform chemical structure with 100% selectivity. Thus, prepared GNRs show well-defined electronic and optical properties, which are fully determined by their specific structure and can be further tuned by modulation of their width and edge configuration^10^,^15^,^16^ as well as by atomically controlled doping^12^,^17^. All of this holds promise for application in next-generation optoelectronic and photonic devices, as recently suggested by the realization of all-GNR-based heterojunctions^12^,^18^ and by other proposals for photovoltaic applications^16^,^19^,^20^.

In spite of this interest, the field of GNRs is still in its infancy and little is known about their photophysical properties, especially in the non-equilibrium regime. Extraordinary optical properties were predicted^21^,^22^,^23^, such as width-dependent bandgap and the formation of excitons with extremely high binding energies, which have been only recently demonstrated in bottom-up GNRs^15^,^24^,^25^,^26^. In particular, the pronounced excitonic effects^26^ are accompanied by a significant increase of the optical absorbance, as compared with graphene, for light that is linearly polarized along the ribbon axis. At this stage, the understanding of the excited-state relaxation dynamics of GNRs would offer not only a deeper insight into the fundamental physics of these ideal one-dimensional systems but also a benchmark for their integration in advanced optoelectronic devices^27^.

In the following, we apply resonant ultrafast pump–probe spectroscopy to nanometre-wide atomically precise GNRs obtained by a bottom-up solution synthesis^8^ to study the kinetics of excitons and their interactions in the saturation (that is, nonlinear) excitation regime. For high-excitation fluences, we observe bimolecular exciton annihilation and the concomitant ultrafast (≈1 ps) buildup of a stimulated emission (SE) signal from an excited biexciton state that is populated via a nonlinear process, a result that is extremely promising in view of applications of GNRs as tuneable active materials in lasers and light-emitting diodes.

## Results

### Linear absorption of GNRs

**Figure 1:**
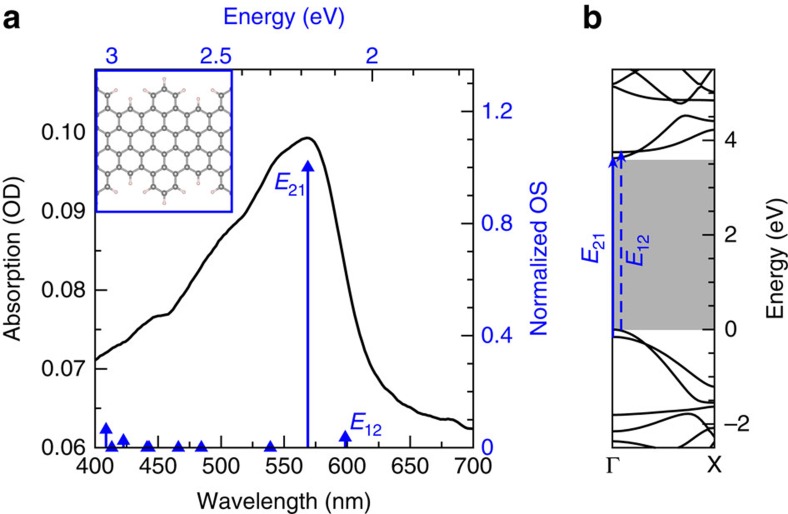
Linear absorption and excitons in GNRs. (**a**) Linear absorption spectrum of the 4CNR sample in THF solution (black curve). A ball-and-stick model of the GNR without alkyl chains at the edges is shown in the inset. The experimental spectrum is compared with the result of *GW*-BS calculations, with excitonic transitions indicated by blue arrows. (**b**) *GW* quasi-particle band structure. The lines indicate the transitions that are mainly contributing to the first and second exciton. The 1.5 eV difference between the *GW* gap (**b**; grey area) and the excitonic transition reported in **a** defines the exciton binding energy in vacuum.

The GNRs studied here, characterized by cove-shaped edge morphology and hereafter labelled 4CNR (following the notation in ref. 16), were chemically synthesized as described in ref. 8. Their aromatic core structure, displayed in Fig. 1a (inset), features a modulated width of 0.7–1.1 nm, and is functionalized with long and branched alkyl chains (2-decyltetradecyl) at the outer benzene rings to guarantee dispersibility in organic solvents. The linear absorption spectrum of the GNR sample in tetrahydrofuran (THF) dispersion is shown in Fig. 1a (black curve), and compared with the simulated gas-phase spectrum obtained from *ab initio GW* plus Bethe–Salpeter (*GW*-BS) calculations (blue arrows), performed on H-passivated 4CNR (details concerning the calculations are reported in the Methods section). THF was chosen as a solvent to minimize aggregation of GNRs, which would significantly alter their optical properties. The effect of the solvent on the spectrum is instead expected to be minor (see, for example, ref. 28). The experimental spectrum is dominated by an optical transition of excitonic origin located at ≈570 nm, in good agreement with simulations. According to our *GW*-BS results, the first two excitons both arise from the linear combination of transitions among the two highest valence and two lowest conduction bands around the *Γ* point (mainly *E*_12_ and *E*_21_ transitions for the first and second exciton, respectively, as indicated in the band structure of Fig. 1b). In vacuum, excitons are tightly bound, with a giant binding energy of ∼1.5 eV (defined as the difference between the quasi-particle gap and the energy of the excitonic states), which is expected to diminish in presence of a dielectric environment.

### Ultrafast pump–probe spectroscopy of GNRs

**Figure 2:**
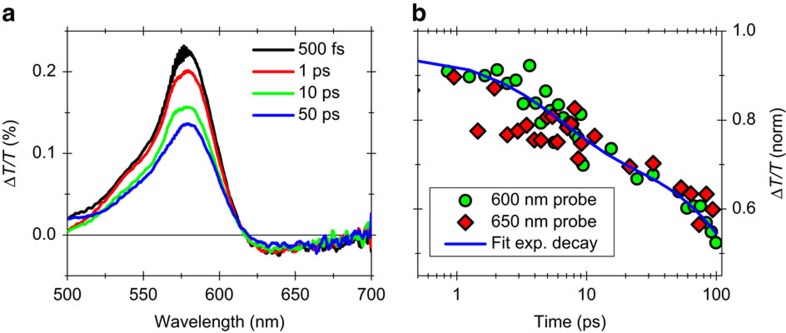
Transient absorption spectra and dynamics at low fluence. (**a**) Δ*T*/*T* spectra of 4CNRs at different pump–probe delays and (**b**) decay dynamics at 600- (green circles) and 650- (red diamonds) nm probe wavelengths for an excitation fluence of ≈100 μJ cm^−2^. The fit (blue line) in **b** correspond to a bi-exponential function with time constants *τ*_1_≈6 ps and *τ*_2_≈330 ps.

We performed broadband pump–probe spectroscopy of the 4CNR samples using resonant excitation at 570 nm and white-light probing covering the 500–700 nm range, with an overall temporal resolution of ≈100 fs (see Methods for details of the experimental setup). Figure 2 shows the differential transmission (Δ*T*/*T*) spectra for different pump–probe delays (Fig. 2a), and the Δ*T*/*T* dynamics at 600 nm probe wavelength (Fig. 2b), when the sample is excited with a low fluence of ≈100 μJ cm^−2^. In the Δ*T*/*T* spectra we clearly distinguish two bands: (i) an intense photo-bleaching (PB) of the excitonic transition peaked at ≈570 nm, which we assign to ground-state depletion and/or phase space filling of the excited state; (ii) a relatively weak red-shifted photo-induced absorption (PA) band starting from ≈620 nm. Since these two bands have the same decay kinetics (Fig. 2b), we attribute the PA signal to excited-state absorption from the exciton to higher energy states or to the e–h continuum. The comparison of the Δ*T*/*T* spectra (Fig. 2a) for different pump–probe delays (from 500 fs to 50 ps) also highlights that the signal decays without any significant spectral evolution at this excitation fluence, thus indicating a simple relaxation mechanism of the excited state (either radiative via emission of photons and/or non-radiative via interaction with phonons).

To understand the carrier relaxation process, we can take as a reference the large amount of experimental results on SWNTs, since we expect them to display similar recombination dynamics. SWNTs also show complex, multi-component decay kinetics. In the low-excitation fluence regime, the long-lived (>5 ps) decay components in SWNTs have been reproduced with a bi-exponential model, which includes both the radiative and non-radiative lifetime^29^,^30^,^31^,^32^. Other studies describe these decay dynamics by a model for a diffusion-limited regime^33^,^34^ or geminate e–h recombination in one dimension^35^, thus leading to a power law (≈*t*^−0.5^) kinetics. Since a detailed study of the dynamics of these long-lived photoexcitations for GNRs is beyond the scope of this paper, we fit the kinetics of the PB signal at 600 nm probe wavelength with a simple bi-exponential decay model (Fig. 2b), which gives us the timescale of the relaxation processes. From the fit we obtain *τ*_1_≈6 ps and *τ*_2_≈330 ps, in good agreement with the results obtained for SWNTs^30^.

**Figure 3:**
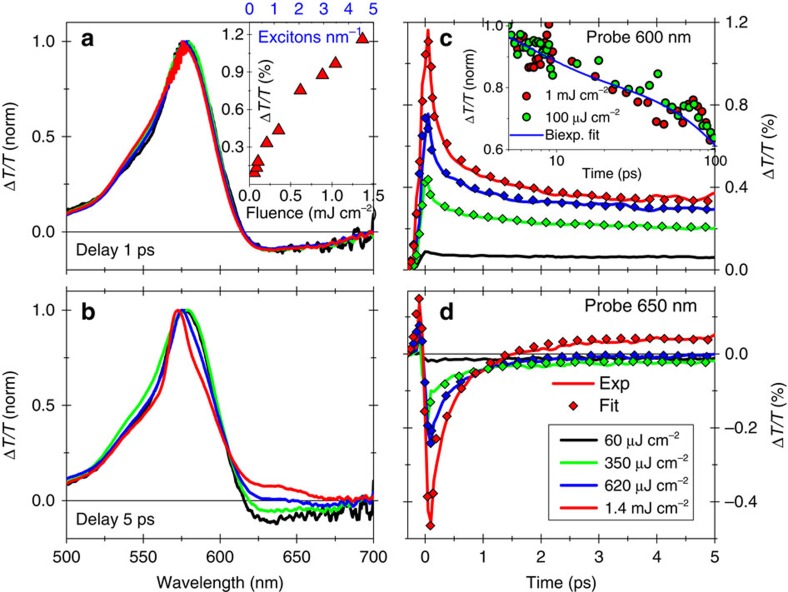
Exciton–exciton annihilation and biexciton formation. Normalized Δ*T*/*T* spectra of 4CNRs for different excitation fluences at a fixed pump–probe delay of (**a**) 1 ps and (**b**) 5 ps. The inset in **a** reports the peak amplitude of the signal at 600 nm probe wavelength as a function of the excitation fluence (bottom *x* axis) and the exciton linear density (top *x* axis). Excitation-fluence-dependent dynamics at (**c**) 600 nm probe wavelength and (**d**) 650 nm probe wavelength. The fit (diamonds) is obtained from the coupled rate-equations (described in the text) based on exciton–exciton annihilation in one dimension. Inset in **c** represents the dynamics on a 100 ps timescale for 100 μJ cm^−2^ (low) and 1 mJ cm^−2^ (high) fluences, together with the bi-exponential fit used in Fig. 2b.

Instead, we concentrate on the ultrafast (<5 ps) decay dynamics, and in particular on their dependence on the excitation fluence, as we show in Fig. 3. The normalized Δ*T*/*T* spectra at different excitation fluences for a 1 ps pump–probe delay (Fig. 3a) display very similar, fluence-independent PB and PA spectral features, while for a 5 ps pump–probe delay (Fig. 3b) we unambiguously observe the buildup, with increasing fluence, of a positive and red-shifted Δ*T*/*T* peak, at ≈650 nm. From a general point of view, a positive Δ*T*/*T* signal in pump–probe experiments describes either a PB or a SE process. We here assign the peak at ≈650 nm to a SE process, since (i) it does not correspond to any resonant feature in the linear absorption spectrum (Fig. 1a), as also confirmed by simulations, being instead red-shifted with respect to the main excitonic transition (at ≈570 nm); and (ii) it appears with a ≈ps delay with respect to the pump pulse and only at high-excitation fluences. We note that photons produced by SE are identical (phase, energy and momentum) to the probe photons and thus can be detected in pump–probe experiments, at variance with those produced by spontaneous emission.

### Exciton–exciton annihilation and biexciton formation in GNRs

To clarify the origin of this SE signal, we concentrate on the fluence-dependent dynamics at the probe wavelengths of 600 nm, that is, the PB signal, and 650 nm, that is, the SE signal (Fig. 3c and d, respectively). We immediately observe that, for increasing fluence, the PB signal (Fig. 3c) displays a faster decay, while, correspondingly, the signal at 650 nm (Fig. 3d) undergoes a clear change in sign (from negative to positive) that corresponds to the delayed formation of the SE signal. First, let us analyse the fast PB decay at 600-nm probe wavelength (Fig. 3c). In semiconducting SWNTs the appearance of an ultrafast fluence-dependent decay component is explained by exciton–exciton annihilation, a two-exciton interaction process, in which one exciton recombines to the ground state and the other either dissociates into a free e–h pair or is promoted into a higher energy level^36^,^37^,^38^. Such process is also commonly observed in other one-dimensional semiconductors, such as conjugated polymers^39^,^40^, and it has been recently observed also in monolayer MoS_2_ (ref. 41). Theoretical calculations^42^ also predict that an Auger-like mechanism occurs in semiconducting armchair GNRs because of effectively enhanced Coulomb interaction. In their work, Konabe *et al*.^42^ find an exciton–exciton annihilation time in the order of few ps for 1.2–2.5 nm wide GNRs. After the initial ultrafast nonlinear decay process, the dynamics at all pump fluences are instead the same. This can be noticed by comparing the kinetics of the PB signal at high and low fluences over the full temporal range (inset in Fig. 3c). Being a two-body interaction process, exciton–exciton annihilation is expected to display a nonlinear dependence on the exciton density (see Methods for details) and/or the excitation fluence^37^, in agreement with our experimental results (inset in Fig. 3a).

**Figure 4:**
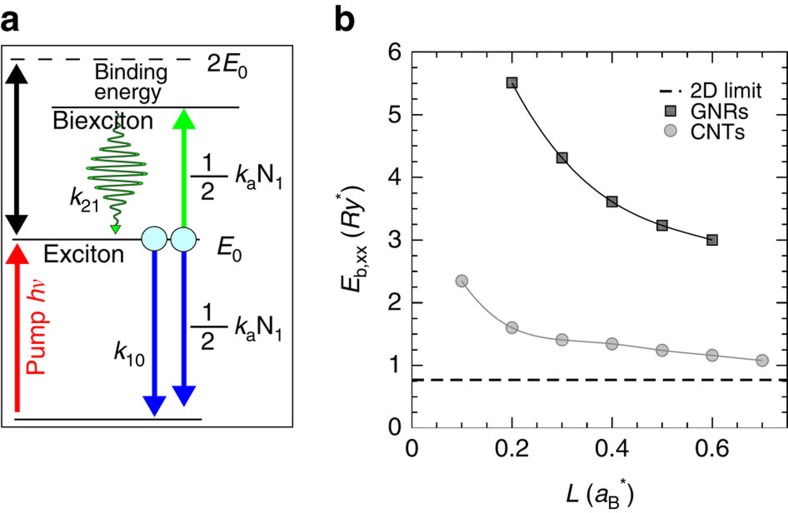
Photoexcitation scenario in GNRs and biexciton binding energy. (**a**) Sketch of the energetic levels and the kinetic model described in the text. (**b**) Biexciton (XX) binding energy (*E*_b,XX_) as a function of the lateral dimension (*L*), that is, width for GNRs (black squares) and diameter for SWNTs (grey circles). The data, obtained by guide-function QMC simulations, are shown in dimensionless exciton units, as detailed in the text. In these units, the binding energy of the 4CNR is ∼3.4 Ry*. The dashed line indicates the two-dimensional value of *E*_b,XX_=0.77 Ry*.

Second, we need to understand the origin of the delayed formation of the SE signal at 650 nm (Fig. 3d). As we have already discussed, following exciton–exciton annihilation both free e–h pairs and/or higher energy-excited states can be formed. The first scenario has been observed in SWNTs, where the creation of charges in the high-excitation regime leads to the formation of trions^43^,^44^, which are detected as a negative (PA) and red-shifted Δ*T*/*T* signal. Clearly, this scenario is in contrast with our experimental results, which present a positive (SE) signal. Instead, the formation of a delayed and red-shifted SE signal in the high-excitation regime was observed in semiconducting quantum-dots (QDs)^45^,^46^,^47^,^48^, another prototype of quantum-confined systems. In the case of QDs, the SE signal was explained in terms of emission from biexcitons and the energy distance between the main exciton PB signal and the biexciton SE signal gives the biexciton binding energy, which is typically of the order of few tens of meV. For our GNRs, the SE peak corresponds to a much larger value for the biexciton binding energy, that is, *E*_b_≈250 meV. This value is in accordance with the comparably larger exciton binding energies in GNRs, that are at least one order of magnitude larger than in QDs due to the reduced screening as well as the extreme two-dimensional and transversal confinements. The following photoexcitation scenario in GNRs thus emerges: at high-excitation fluences, exciton–exciton annihilation leads to the population of a radiative biexciton state, which then undergoes SE to the one-exciton level upon interaction with the probe pulse (Fig. 4a).

## Discussion

To support our assignment, we first compute the biexciton binding energy in GNRs by means of guide-function quantum Monte Carlo (QMC) simulations, using an envelope function approach with effective masses, as previously reported in ref. 49 for SWNTs. Full description of the method is reported in the Methods section. In Fig. 4b, we show the biexciton binding energy dependence on the lateral confinement in dimensionless exciton units, that is, distances are measured in units of the effective Bohr radius 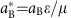 and energies in units of the effective Rydberg 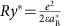, where *μ* is the reduced e–h mass, *ɛ* is the dielectric constant, *a*_B_ is the Bohr radius and *e* is the electron charge. By considering an average reduced mass as obtained from *ab initio* calculations (computed as the weighted average relative to the *E*_21_ and *E*_12_ transitions, that is, *μ*=0.22), the average width of the 4CNR (*w*=0.84 nm), and the dielectric constant of the solvent (*ɛ*=7.5), we obtain a biexciton binding energy of 180 meV, in good agreement with the experimental result of 250 meV. The discrepancy with respect to experiments is reasonable in view of the simplified description scheme used here, which is not expected to capture all microscopic details.

In Fig. 4b, we also report the biexciton binding energy for SWNTs, calculated by the same approach^49^: for a SWNT of similar lateral dimension, we find a binding energy that is less than one-half of that of the 4CNR in dimensionless units. This can be understood by considering the different biexciton confinement, since in the case of SWNTs the biexciton wavefunction is delocalized over the whole circumference, whereas in GNRs it becomes strongly confined in the transversal direction. The value obtained for GNRs is quite large also in comparison with the biexciton binding energy *E*_b_≈50 meV of different monolayer transition metal dichalcogenides obtained by recent transient absorption^50^ and photoluminescence experiments^51^.

To further confirm our interpretation, we finally reproduce the temporal evolution of the exciton (that is, decay of the PB signal) and the biexciton (that is, formation of the SE signal) populations with the following coupled rate-equations^52^,^53^,^54^:


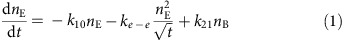



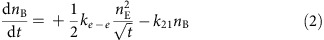


where *n*_E_ and *n*_B_ are the exciton and biexciton population, respectively, *k*_10_ is the decay rate from the exciton to the ground state, *k*_21_ is the radiative decay rate from the biexciton to the exciton producing the SE signal and *k*_*e–e*_ is the exciton annihilation rate constant (see Fig. 4a for the adopted model). The *t*^−0.5^ dependence of the exciton annihilation rate arises from the one-dimensional diffusion mechanism of excitons, and its divergence for *t*→0 is cured by truncating it for times shorter than the width of the instrumental response function (IRF) (≈100 fs in our case). The quality of the fit (Fig. 3c,d) further confirms the validity of our model. From the fit we find that *k*_10_≈0 ps and *k*_21_≈0.15 ps^−1^, meaning that both mechanisms occur on a timescale that is longer with respect to the temporal window (5 ps) that we use for this analysis.

It is worth noting that we can fit our experimental data considering that all the excitons that undergo exciton–exciton annihilation form biexcitons. In particular, we can exclude that the observed SE signal is because of the dissociation of excitons into free e–h carriers, since SE from electrons in the continuum is not expected. Another possibility is that free e–h pairs get trapped into low-energy states; for example, due to defects. Nevertheless, since the selection rules for emission and absorption of photons are the same, the presence of such bright low-energy states should be detectable also in the linear absorption spectrum, and thus this scenario can be also excluded by looking at the absorption spectrum and theoretical simulations in Fig. 1. Thus, although we cannot exclude that free e–h carriers are formed, we can strongly assert that the observed SE arises from biexcitons. Since our model is able to correctly reproduce also the amplitude of the pump–probe signal without additional loss channels, we conclude that biexciton formation upon exciton–exciton annihilation is extremely efficient. Finally, for a fixed pump–probe delay we can also evaluate the annihilation rate 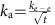, which corresponds to an initial *k*_a_≈2 ps^−1^ for a pump–probe delay of 180 fs, close to our temporal resolution, and thus to an annihilation lifetime of ≈0.5 ps, in excellent agreement with experimental results on SWNTs^54^ and theoretical calculations on GNRs^42^.

In conclusion, we studied the transient photophysical properties of ultranarrow structurally well-defined GNRs by means of ultrafast pump–probe spectroscopy. We show that a nonlinear decay channel for the main excitonic transition sets in at high excitation densities, and, correspondingly, we unambiguously observe a red-shifted SE signal. Our experiments demonstrate that exciton–exciton annihilation populates a radiative biexciton state, with an extremely high binding energy ≈250 meV, in agreement with estimates from QMC simulations. The high efficiency we find for both exciton–exciton annihilation and biexciton formation is of great importance, not only for gaining fundamental understanding on strongly enhanced quantum effects in low-dimensional materials but also for its implications in GNR-based optoelectronic devices. Indeed, the clear observation of a strong SE is extremely promising in view of using GNRs as active light-amplifying materials in tuneable lasers and light-emitting diodes. Moreover, our results suggest that also multiple-exciton generation^55^,^56^,^57^ can be extremely efficient in GNRs since it is governed by the same exciton–exciton annihilation rate. A detailed understanding of the photophysics of biexcitons and the mechanism of multiple-exciton generation will help to improve the efficiency of photovoltaic devices, with GNRs acting as light absorbers.

## Methods

### Pump–probe experimental setup

The experimental setup used for pump–probe measurements has been described in detail elsewhere^58^. In brief, the setup is based on a regeneratively amplified Ti:sapphire laser (Coherent, Libra) producing 100 fs, 4 mJ pulses at 800 nm wavelength and 1 kHz repetition rate. The probe pulse is obtained by focusing a fraction of the laser beam in a 2-mm-thick sapphire plate to generate a broadband single-filament white-light continuum. The pump pulse, generated by an optical parametric amplifier, is centred at 570 nm (at resonance with the main excitonic transition) with a bandwidth of ≈10 nm, corresponding to ≈70 fs duration. The probe light transmitted by the sample is dispersed on an optical multichannel analyser equipped with fast electronics, allowing single-shot recording of the probe spectrum at the full 1 kHz repetition rate. By changing the pump–probe delay, we record two-dimensional maps of the differential transmission (Δ*T*/*T*) signal as a function of probe wavelength and delay. The temporal resolution (taken as full-width at half-maximum of pump–probe cross-correlation) is ≈100 fs over the entire probe spectrum.

### Exciton linear density estimation

To estimate the exciton linear density reported in the inset of Fig. 3a, we proceed as follows: (i) we calculate the number of absorbed photons cm^−2^ from the measured pump fluence, the measured absorbance, and the pump photon energy (2.18 eV); (ii) we calculate the number of excitons cm^−2^ by considering an exciton photogeneration efficiency (Quantum Yield) of 96% based on optical pump—THz probe experiments on similar GNRs^59^; and (iii) we multiply this value by the concentration (0.0021, g l^−1^) of the dispersion and divide by the mass of a unit length of the GNRs (0.83 × 10^−21^ g nm^−1^). This estimate gives a density of ∼0.2–0.3 excitons nm^−1^ for pump fluences below 100 μJ cm^−2^, corresponding to an average distance between excitons of ∼4–5 nm. Such a value appears to be sufficient to prevent exciton–exciton interactions.

### Coherent artefacts in pump–probe dynamics

The coupled-rate equations used to model the evolution of the exciton and biexciton populations are solved by taking into account both the IRF and possible coherent artefacts present in pump–probe measurements. For our experimental setup the IRF is the cross-correlation of the pump and probe pulses, and can be quite accurately modelled by a Gaussian function with ≈100 fs full-width at half-maximum. Possible coherent artefacts in pump–probe experiments are stimulated Raman amplification and cross-phase modulation (XPM)^60^. We fitted the initial 100 fs of the PB signal at 600 nm (Fig. 3c) including the IRF to reproduce the initial buildup and a Gaussian function (the same used for the IRF) to reproduce the initial ultrafast stimulated Raman amplification coherent artefact. For the SE dynamics at 650 nm (Fig. 3d), instead, we include also XPM, which we fit as the first derivative of a Gaussian function (the same used for the cross-correlation). Although extremely simple, this model correctly reproduces not only the evolution but also the initial steps of the pump–probe signal.

### Thermal effects and sample heating

Time-resolved experiments were carried out at room temperature, assuming negligible temperature effects on the spectra on the basis of previous results on SWNTs (see, for example, ref. 61). Regarding the sample heating during experiments, we also expect negligible temperature changes upon photoexcitation. In fact, we can estimate a maximum increase in temperature of ∼0.1 K, based on a comparison with the work of Abdelsayed *et al*.^62^, and by considering the following parameters: volume of the sample (0.3 ml), THF heat capacity (123 J mol^−1^ K^−1^), density (889 kg m^−3^), molar mass (72 g mol^−1^) and laser total energy (∼100 nJ per pulse at 500 Hz for 10 min of irradiation, corresponding to 30 mJ). This indicates that we are working in a perturbative regime for what concerns thermal effects.

### *GW*-BS calculations for the static absorption

The ground-state atomic structure of the 4CNR was optimized by using the PWscf code of the Quantum ESPRESSO package (ref. 63), which is based on a plane-wave pseudopotential implementation of density functional theory. Calculations were performed by employing local density approximation exchange correlation (xc) functional and norm-conserving pseudopotentials, with a cutoff energy for the wavefunctions of 60 *Ry*. The atomic positions within the cell were fully relaxed until forces were <5 × 10^−4^ *Ry* bohr^−1^, while the lattice constant along the periodic direction was optimized separately. The Brillouin zone was sampled with 16 **k**-points along the periodic direction. The Kohn-Sham band structure obtained for the optimized geometry was improved by introducing many-body corrections within the *G*_0_*W*_0_ approximation for the self-energy operator. Here, the dynamic dielectric function was obtained within the plasmon-pole approximation, by employing a box-shaped truncation of the Coulomb potential^64^ to remove the long-range interaction between periodic images. The optical absorption spectrum was then computed as the imaginary part of the macroscopic dielectric function starting from the solution of the BS equation, which allows for the inclusion of e–h interaction effects. The static screening in the direct term was calculated within the random-phase approximation with inclusion of local field effects; the Tamm–Dancoff approximation for the BS Hamiltonian was employed. The aforementioned *GW*-BS calculations, performed by using the YAMBO code (ref. 65), were carried out for the fully H-passivated 4CNR, that is, by removing the alkyl side chains, in order to make them computationally affordable. We have checked that the different passivation does not affect the band structure properties at the density functional theory and local density approximation level in the energy window of interest for the determination of the optical absorption. Similar results were reported in ref. 66.

### The guide function quantum Monte Carlo approach

Atomistic simulations as the ones described above are presently unfeasible for the investigation of biexcitons in realistic systems. Here we resort to an effective model based on guide function QMC simulations, as previously reported in ref. 49 for SWNTs. For the (unnormalized) guide function we use


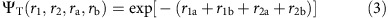


where *r*_1,2_ (*r*_a,b_) are the dimensionless positions of the two electrons (holes), and 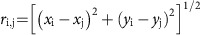 is the distance between particles confined to the two-dimensional nanoribbon. The Monte Carlo simulation approach is the same as in ref. 49 with the only exception that we add a box-like confinement potential


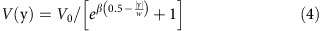


along the transversal direction, with *V*_0_=1,000, *β*=20 and *w* the GNR width. In the simulations we use 20,000 walkers^67^, a time step of Δ*t*=0.25 × 10^−4^, an equilibration interval of 20,000 and a measurement interval of 30,000 time steps. The smoothed confinement potential and the time step were chosen such that for ‘typical' paths the inequality 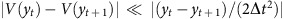 holds^68^. We checked that the biexciton binding energy did not change substantially upon modifying Δ*t* or other simulation parameters. The QMC approach has been able to predict quite accurately the biexciton binding energy of SWNTs. In fact, Colombier *et al*.^69^ detected the presence of biexcitons in SWNTs embedded in a gelatine matrix by means of nonlinear optical spectroscopy, reporting a binding of 106 meV energy for the (9, 7) tube. The QMC model predicts a value of the binding energy of ≈100 meV assuming a dielectric constant of *ɛ*=3 (instead of 2.3 as appropriate for a gelatin matrix). Such an overestimation in the case of small values of *ɛ* is known for phenomenological models, whereas for larger values of *ɛ* (like the one considered in this work, i.e., *ɛ*=7.5) the QMC model is expected to give results similar to more refined (though not yet atomistic) approaches, as discussed in ref. 70.

## Additional information

**How to cite this article:** Soavi, G. *et al*. Exciton-exciton annihilation and biexciton stimulated emission in graphene nanoribbons. *Nat. Commun.* 7:11010 doi: 10.1038/ncomms11010 (2016).
